# Docosahexaenoic Acid (DHA) Decreases IL-6 and Prostaglandin-Endoperoxide Synthase 2 mRNA Expression and IL-6 Protein Release, While Increasing Resolvin D1 and CXCL8 mRNA Expression and Protein Release in BovineEndometrial Cells

**DOI:** 10.3390/ani15172545

**Published:** 2025-08-29

**Authors:** Gisselle Sanchez, Noemi Gutierrez, Mauricio Moya, Rafael A. Burgos, Maria A. Hidalgo

**Affiliations:** Institute of Pharmacology and Morphophysiology, Faculty of Veterinary Sciences, Campus Isla Teja s/n, Universidad Austral de Chile, Valdivia 5090000, Chile; gisselle.sanchez@alumnos.uach.cl (G.S.); noemisgb@gmail.com (N.G.); mauricio.moya@alumnos.uach.cl (M.M.); rburgos1@uach.cl (R.A.B.)

**Keywords:** endometrial cell, docosahexaenoic acid (DHA), resolvin D1, inflammation, cows

## Abstract

The endometrial immune response in postpartum cows is key to maintaining uterine health and preventing inflammatory diseases such as metritis and endometritis. Appropriate management strategies and diets that enhance the immune response are crucial during the transition period; therefore, diets rich in omega-3 fatty acids have been proposed for their potential beneficial effects on cows. Docosahexaenoic acid (DHA) is an omega-3 fatty acid with anti-inflammatory effects in immune cells; however, its effects on bovine endometrial immunity are not fully known. This study aimed to determine the effect of DHA on the inflammatory response in bovine endometrial (BEND) cells. BEND cells were incubated with DHA without or with lipopolysaccharide (LPS), and the mRNA expressions of prostaglandin-endoperoxide synthase 2 (PTGS2), interleukin (IL)-6, and CXCL8 were analyzed using RT-qPCR. The protein amount of IL-6, or CXCL8, and Resolvin D1 (RvD1) in the cell culture medium were analyzed using ELISA. DHA significantly reduced the expression of LPS-induced IL-6 and PTGS2 but increased LPS-induced CXCL8 expression. In addition, DHA reduced LPS-induced ERK1/2 and Akt phosphorylation, as assessed by immunoblotting. DHA increased the production of RvD1, a metabolite of DHA, at 8 and 24 h. In addition, RvD1 reduced LPS-induced CXCL8 production and increased the phosphorylation of ERK1/2 and Akt. Finally, changes in metabolite levels, such as an increase in 2-hydroxypyridine in DHA-treated cells, were obtained using a metabolomic assay. In conclusion, DHA reduced IL-6 and PTGS2 mRNA expression and IL-6 protein release and increased RvD1 levels in bovine endometrial cells, which suggest that DHA could have beneficial effects on endometrial immunity. The increase in CXCL8 mRNA expression and protein release induced by DHA remains to be studied; however, it could play a role in the innate defensive mechanisms of phagocytes.

## Simple Summary

Uterine inflammatory diseases, such as metritis or endometritis, in postpartum cows are a frequent health problem in dairy farms, with consequences on fertility, milk production, and treatment costs. Uterine immunity may be affected by changes that occur during the transition period in dairy cows; therefore, appropriate management strategies and a diet that enhances the immune response are crucial. Omega-3 rich diets have been proposed for their potential beneficial effects on cows, as they can improve reproductive parameters. Docosahexaenoic acid (DHA) is an omega-3 fatty acid mostly found in fatty fish and algae, with recognized anti-inflammatory effects in immune cells; however, its effects on bovine endometrial immunity are not clear. In this study, we used a bovine endometrial cell line and demonstrated that DHA reduced the production of some pro-inflammatory mediators and increased the amount of a chemoattractant for phagocytes. Also, DHA stimulated the production of resolvin D1, a pro-resolving mediator. In addition, DHA reduced the activation of signaling pathways involved in cellular activation. We also performed a metabolomics assay and detected changes in some metabolites that could affect inflammation. In conclusion, this study showed that DHA triggers anti-inflammatory and pro-resolving conditions in bovine endometrial cells but also increases the expression of a chemotactic factor, suggesting that DHA could have beneficial effects on postpartum endometrial immunity, preventing or improving uterine diseases in postpartum cows.

## 1. Introduction

Uterine inflammatory diseases in post-calving cows are a frequent health problem for dairy farms, with consequences on fertility, milk production, and treatment costs. Uterine immunity may be affected by changes that occur during the transition period in dairy cows. Diverse studies have identified risk factors that lead to negative energetic balance (NEB) and can impair immune function [1,2]. NEB, driven by decreased dry matter intake, elevated nutrient requirements for milk production, and high energy demand, leads to intensified lipolysis and elevated levels of non-esterified fatty acids (NEFA) in the blood. Increased NEFA concentrations have been linked to impaired immune cell function and heightened inflammation [1,3]. NEFA can promote innate immunity, production of cytokines, proteases, acute phase proteins, and mitochondrial reactive oxygen species (ROS), and activate intracellular signaling pathways, triggering an inflammatory response [1,4]. Approaches to modulating inflammation have proposed that stress control through appropriate management strategies and a diet that enhances the immune response could be key during the transition period and post-calving.

Dietary supplementation with omega-3 fatty acids has been proposed for its potential beneficial effects in dairy cows because it can favor an anti-inflammatory state. Although a proinflammatory state is characteristic of initial uterine involution to eliminate the massive peripartum influx of pathogens, it must switch to an anti-inflammatory or pro-resolving state to restore endometrial homeostasis and prevent chronic inflammation [5]. Bacterial uterine infections are mostly caused by Gram-negative *Escherichia coli* and Gram-positive *Trueperella pyogenes* [6]. Lipopolysaccharide (LPS) from Gram-negative bacteria is recognized by Toll-like receptor 4, which is expressed in endometrial cells, and induces signaling pathways and the secretion of IL-1β, IL-6, CXCL8, and prostaglandin E2 (PGE2) [7,8]. Numerous studies have assessed the impact of omega-3 fatty acid supplementation on the reproductive and productive performance and health of postpartum cows, showing beneficial effects on fertility and a decrease in the incidence of diseases [9,10,11,12,13].

In addition, a diet rich in omega-3 fatty acids attenuated the acute phase response after an intramammary challenge with LPS and reduced the liver inflammatory response, suggesting an effect on attenuating inflammatory responses [14,15]. However, supplementation with omega-3 enriched fat at 11 days in milk (DIM), which improved reproductive parameters, such as conception rate to first AI and calving-to-conception interval, but did not affect endometrial inflammatory status [16]. Furthermore, dietary omega-3 fatty acid supplementation decreased the expression of genes encoding antigen presentation, complement components, and transcription factors in the endometrium of cows and heifers [9,17,18]. Therefore, further studies are necessary to clarify in more detail the role of omega-3 fatty acids in bovine endometrial immunity.

Docosahexaenoic acid (DHA) is an omega-3 polyunsaturated fatty acid (PUFA) with recognized immunomodulatory and beneficial effects on health and diseases [19]. DHA is mostly found in marine sources, such as fatty fish and algae, but also in vegetable oils and flaxseeds, and it is usually obtained by dietary intake or as a nutritional supplement [20]. Several studies have determined the effects of DHA on immune cells and their possible mechanisms of action. DHA shows antiproliferative effects and reduces the migration and expression of IL-2 and interferon-γ in T-cells [21,22,23,24,25]. Furthermore, DHA reduces the production of tumor necrosis factor-alpha (TNF-α), IL-6, monocyte chemoattractant protein-1, and IL-1β, as well as inflammasome activation in murine macrophages [22,26]. In bovine neutrophils, DHA induces matrix metalloproteinase (MMP)-9 and ROS production [27], suggesting that neutrophil-mediated innate immunity is enhanced. In contrast, DHA inhibits LPS-activated PGE2 production in bovine endometrial cells [28]. The anti-inflammatory and pro-resolving mechanisms that could explain the effects of DHA on immune cells include the interference with activation of the Toll-like receptor 4 (TLR4)/TAK1/Nuclear Factor-kappaB (NF-κB) pathway through the Free Fatty Acid-4 (FFA4) receptor and the synthesis of specialized pro-resolving mediators (SPM) of the D-series resolvin (RvD) [29,30].

In bovines, the FFA4 receptor has been described in neutrophils, ovarian follicles, and endometrial cells [27,28,31]. However, the effects of DHA on both MMP-9 and ROS release in bovine neutrophils and LPS-activated PGE2 production in bovine endometrial cells are independent of the FFA4 receptor, because a selective FFA4 receptor antagonist did not reduce these effects [27,28], suggesting that other mechanisms might mediate these responses in these bovine cells.

DHA can be converted to D-series resolvins (RvD1-6) through reactions that begin with the lipoxygenation of DHA mediated by the enzyme 15-lipoxygenase (15-LO), and depending on the biosynthetic pathway, different resolvins can be produced [32]. In turn, RvD1 activates both ALX/FPR2 and GP32 receptors and enhances macrophage phagocytosis [32,33]. The role of resolvins in inflammation and immune cells has been extensively studied. In contrast, the role of RvD1 in the endometrium has only been proposed for decidualization, showing that RvD1 reduces decidualization biomarkers in human endometrial stromal cells [34]. Likewise, SPM are involved in pregnancy progression because dysregulated SPM levels have been observed in pregnancy complications [35].

In this study, we aimed to determine the anti-inflammatory effect of DHA in bovine endometrial cells by determining LPS-induced IL-6, CXCL8, and prostaglandin-endoperoxide synthase 2 (PTGS2) levels; LPS-induced ERK1/2 and Akt phosphorylation; RvD1 production, a metabolite derived from DHA; and metabolomic changes using metabolomic assays.

## 2. Materials and Methods

### 2.1. Cell Culture of Bovine Endometrial (BEND) Cells

Bovine endometrial (BEND) cells (ATCC^®^ CRL2398™), a cell line derived from the uterine endometrium of a cow on d14 of the estrous cycle [36], were obtained from ATCC (Manassas, VA, USA). BEND cells were cultured according to the manufacturer’s protocol and as described by Valenzuela et al. [28]. Prior to each experimental procedure, the culture medium was replaced with a serum-reduced medium (2% serum) devoid of antibiotics, and the cells were maintained for a duration of two hours.

### 2.2. Real Time-qPCR

BEND cells (*n* = 5 independent experiments from different passages) grown in 60 mm plates were incubated with a vehicle (0.1% dimethyl sulphoxide (DMSO)) or 50 μM DHA for 30 min, and then a vehicle (1× phosphate buffered saline (PBS)) or LPS (500 ng/mL) (*Escherichia coli* O111:B4, Sigma-Aldrich, Saint Louis, MO, USA) was added and incubated for 16 h at 37 °C. The DHA concentration used was based on our previous study in BEND cells [28]. The LPS concentration was chosen based on the studies of [8,37]. The time of 16 h was chosen based on a previous time-course study that showed a significant increase in inflammatory gene expression between 1 and 24 h [38]. Total RNA was isolated using the EZNA Total RNA Isolation Kit (Omega Bio-Tek, Norcross, GA, USA), and it was treated with DNase to ensure removal of genomic DNA. One microgram of total RNA was used for cDNA synthesis with Affinity Script cDNA Synthesis Kit (Agilent, Santa Clara, CA, USA). Real-time PCR was performed with primers specific for bovine IL-6, CXCL8, PTGS2, and the housekeeping ribonucleoprotein S9 (RSP9), using the Brilliant II SYBR Green qPCR Kit (Agilent, Santa Clara, CA, USA). The primers were the following: IL-6 F 5′-TCCTGAAGCAAAAGATCGCA-3′ and R 5′-CCCACTCGTTTGAAGACTGC-3′; CXCL8 F 5′-AAACGAGGTCTGCCTAAACCC-3′ and R 5′-TCTTGCTTCTCAGCTCTCTTCAC-3′; PTGS2 F 5′-GCATAAGCTGCGCCTTTTCA-3′ and R 5′-CAGGAACATGAGGCGGGTAG-3′; and RSP9 F 5′-GCTGACGCTGGATGAGAAAGACCC-3′ and R 5′-ATCCAGCACCCCGATACGGACG-3′ [39,40]. PCR conditions were the following: 95 °C for 3 min, 40 cycles of 10 s at 95 °C, and 60 s at 60 °C. Post-PCR melting curves were used to confirm the specificity of single-target amplification. The reaction efficiency, determined using the StepOne v2.3 software (Applied Biosystems, Waltham, MA, USA), ranged from 95% to 110% for the primers used. The relative abundance of each gene was calculated relative to RSP9 mRNA using the 2^−ΔΔCt^ method [41], with the formula of relative abundance = 2^(−ΔCt)^, where ΔCt is the difference between the Ct value of each gene (IL-6, CXCL8, and PTGS2) and that of RSP9 [42]. The results are presented as bar graphs, representing the relative expression levels of mRNA for each gene.

### 2.3. IL-6, CXCL8, and RvD1 ELISA Assay

BEND cells (*n* = 3 independent experiments from different passages) were cultured in 24-well plates with a vehicle (0.1% DMSO) or 50 μM DHA for 30 min, and then a vehicle (1X PBS) or LPS (500 ng/mL) was added and incubated for 24 h at 37 °C. A time of 24 h was chosen based on previous studies [8,37]. In other experiments, BEND cells were incubated with 100 nM RvD1 (#12012554, Cayman Chemical, Ann Arbor, MI, USA) for 30 min, and then a vehicle or LPS (500 ng/mL) was added and incubated for 24 h at 37 °C. Supernatants were obtained and stored at −80 °C until the ELISA was performed. IL-6 and CXCL8 were assessed in duplicate using the IL-6 Bovine ELISA Kit (#ESS0029, Thermo Fisher Scientific, Vienna, Austria) and Bovine IL-8 (CXCL8) ELISA BASIC Kit (#3114-1A-6, MABTECH AB, Nacka Strand, Sweden), according to the manufacturers’ instructions. For RvD1 determination, BEND cells were cultured in 24-well plates with a vehicle (0.1% DMSO) or 50 μM DHA for 8 or 24 h at 37 °C. In another experiment, BEND cells were incubated with (0.1% DMSO) or 5 μM ML351 (Cayman Chemical, Ann Arbor, MI, USA), a selective 15-LO inhibitor, for 30 min, and then a vehicle (0.1% DMSO) or 50 μM DHA was added and incubated for 8 h at 37 °C. In a third experiment, BEND cells were incubated with (0.1% DMSO) or 50 μM DHA for 30 min, and then a vehicle (1X PBS) or LPS (500 ng/mL) was added and incubated for 8 h at 37 °C.

### 2.4. Immunoblot

BEND cells cultured in a 60 mm plate (*n* = 3 independent experiments from different passages) were incubated with a vehicle (0.1% DMSO) or 50 μM DHA for 15 min, and then a vehicle (1X PBS) or LPS (500 ng/mL) was added and incubated for 15 min (for ERK1/2 assay) or 60 min (for Akt assay) at 37 °C. In other experiments, BEND cells were incubated with 100 nM RvD1 for 0, 5, 15, 30, or 60 min at 37 °C. The cells were then lysed according to the method described by Gutierrez et al. [40]. Fifty micrograms of protein were separated on 10% SDS–PAGE gels and transferred to a nitrocellulose membrane. Immunoblots were performed according to the protocol recommended by the manufacturer of the phospho-ERK1/2 (# 9101, Cell Signaling, Beverly, MA, USA) or phospho-Akt antibody (# 4060, Cell Signaling, Beverly, MA, USA). Both antibodies were used at a dilution of 1:2000. The bands of phospho-ERK1/2 and phospho-Akt were visualized using an Odyssey Fc infrared/chemiluminescence system (LI-COR Biosciences, Lincoln, NE, USA). Then, the antibodies were removed by incubation in stripping buffer and successive washes. Total anti-ERK1/2 (# 9102, Cell Signaling, Beverly, MA, USA) or anti-Akt antibody (# 9272, Cell Signaling, Beverly, MA, USA) at a dilution of 1:2000 were used to detect total protein levels. Band intensity was measured using the Image Studio Lite v5.2 software (LICOR Biosciences). All antibodies used were reactive to bovine proteins, as indicated by the manufacturer. The results are shown in a representative image of three experiments, and the band intensities are shown in a bar graph as the ratio of phospho-ERK1/2/total-ERK1/2 and phospho-Akt/total-Akt.

### 2.5. Gas Chromatography–Mass Spectrometry (GC–MS) Metabolomics: Sample Preparation and Metabolomics Assay

BEND cells (*n* = 3 independent experiments from different passages) were cultured in 60 mm plates with a vehicle (0.1% DMSO) or 50 μM DHA for 2 h at 37 °C. After rinsing with 0.9% NaCl, the plates were snap-chilled in liquid nitrogen and scraped to collect the biomass. Metabolites were extracted with a chilled acetonitrile:isopropanol:water mixture (37.5:37.5:25, *v*/*v*/*v*) containing 1 mM ribitol as an internal standard, following a protocol adapted from a previous study [43]. The extracts were vortexed and centrifuged (14,000× *g* for 5 min at 4 °C). The supernatant (450 μL) was dried using a vacuum concentrator at 45 °C. The pellet was reconstituted in 50% acetonitrile and 50% water and dried again under vacuum at 45 °C. A fatty acid methyl ester (FAME) C8–C30 mixture was added for the retention index calibration (#400505, Fiehn GC/MS Metabolomics Standards Kit; Agilent Technologies, Santa Clara, CA, USA). The samples were derivatized by methoximation (10 μL methoxyamine in pyridine, 30 °C, 90 min), followed by silylation (90 μL MSTFA with 1% TMCS, 37 °C, 30 min). The derivatized samples were transferred to glass inserts and capped for GC–MS analysis.

The samples (2 μL) were injected in splitless mode on an Agilent 7890B GC coupled to a 5977A Mass Selective Detector (MSD) system (Agilent technologies, Palo Alto, CA, USA) using a DB-5 column (30 m × 0.25 mm × 0.25 μm). The injector was held at 250 °C; helium carrier gas was set to 1 mL min^−1^. The oven program started at 60 °C and ramped at 10 °C min^−1^ to 325 °C (total runtime of 37.5 min). Electron-impact spectra (50–600 *m*/*z*) were acquired at 20 Hz after a 5.9 min solvent delay; the ion source and quadrupole temperatures were 250 °C and 150 °C, respectively. All samples were analyzed within 24 h of derivatization.

Before analyzing the data, the raw MS data (.D files) were converted into an analysis-based file (.ABF) format using the Reifycs ABF Converter (Reifycs, Tokyo, Japan). Peak detection, deconvolution, and alignment were performed using MS-DIAL v2.83. Trimethylsilylated features were annotated against the NIST and Fiehn libraries using FAME-based retention indices and EI spectral similarity. The identification thresholds were as follows: EI similarity ≥ 65%, identification score ≥ 70%, mass tolerance 0.5 Da, and retention time tolerance of 0.5 min [44].

### 2.6. Statistical Analysis

The experiments were analyzed using a one-way analysis of variance and Dunnett’s multiple comparison test (compared with the control or time 0). Normality and homogeneity of variance were assessed using the Shapiro–Wilk or Brown–Forsythe tests, respectively. Comparisons between treatments are shown in each graph with square brackets and significance levels. The results are shown as bar graphs of the mean ± SEM. Statistical significance was set at *p* < 0.05. All analyses were performed using PRISM v10.4.1 (GraphPad, San Diego, CA, USA).

Metabolomic analysis was performed according to a previously published method [43]. All multivariate analyses were statistically analyzed using MetaboAnalyst v6.0 (Xia Lab, McGill University, Canada; http://www.metaboanalyst.ca, accessed on 7 July 2025) according to previously published protocols [45]. Metabolites that were more than 50% below the detection limit or had at least 50% missing values were excluded from the analyses. The metabolite concentrations were normalized to ribitol, an internal standard. To obtain a Gaussian distribution, logarithmic transformation and auto-scaling were performed before statistical analysis [45]. Partial least squares-discriminant analysis (PLS-DA) and variable importance in projection (VIP) scores were determined. A heat map was generated using the Euclidean distance measure and Ward’s clustering algorithm. Metabolites exhibiting significantly different levels (*p* < 0.05) according to the Mann–Whitney test were considered.

## 3. Results

### 3.1. DHA Reduces IL-6 and PTGS2 Expression and ERK1/2 and Akt Phosphorylation and Increases CXCL8 Induced by LPS in BEND Cells

**Figure 1:**
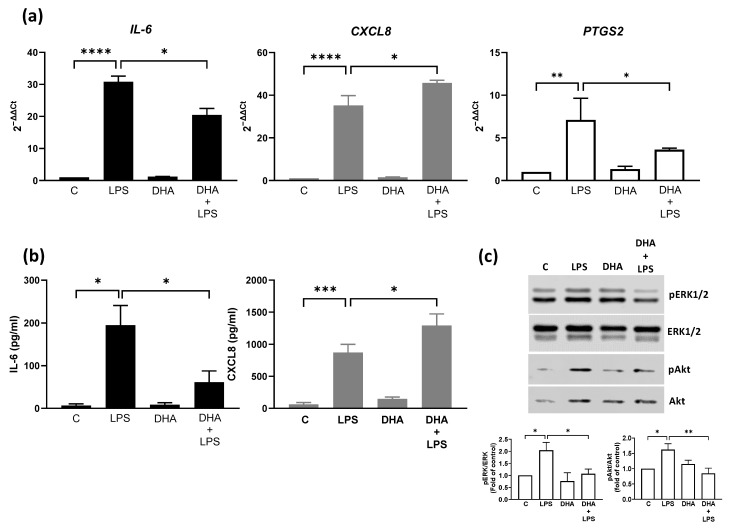
DHA reduced IL-6 and PTGS2 expression and ERK1/2 and Akt phosphorylation and increased CXCL8 expression in BEND cells. BEND cells were incubated with 50 μM DHA (or vehicle), and then 500 ng/mL LPS (or PBS) was added and incubated for 16 h for mRNA expression analysis (**a**) or for 24 h for a ELISA assay in the supernatant (**b**). In (**c**), BEND cells were incubated with 50 μM DHA (or vehicle), and then 500 ng/mL LPS (or PBS) was added and incubated for 15 or 60 min for ERK1/2 or Akt phosphorylation, respectively. Total proteins were analyzed by immunoblotting with antibodies against phospho-ERK1/2, total ERK1/2, phospho-Akt, and total Akt. C = vehicle. Band intensities are shown as the ratio of phospho-ERK1/2/ERK1/2 and phospho-Akt/Akt in the bar graph. Images are representative of three independent experiments. * *p* < 0.05, ** *p* < 0.01, *** *p* < 0.001, **** *p* < 0.0001.

We treated BEND cells with DHA and then with LPS for 16 h to analyze the mRNA expression of PTGS2, il-6, and CXCL8 by RT-qPCR or for 24 h to analyze the production of IL-6 and CXCL8 in the culture medium through ELISA. LPS significantly increased il-6, CXCL8, and PTGS2 mRNA expression, and pretreatment with DHA significantly reduced LPS-induced il-6 and PTGS2 mRNA expression. In contrast, DHA increased LPS-induced CXCL8 mRNA expression (Figure 1a). The production of cytokines in the culture medium showed that DHA significantly reduced LPS-induced IL-6 production but increased LPS-induced CXCL8 production, similar to gene expression (Figure 1b). The effect of DHA on MAPK ERK1/2 and Akt phosphorylation was assessed using immunoblotting. DHA significantly reduced LPS-induced ERK1/2 and Akt phosphorylation in BEND cells (Figure 1c; whole blot images are shown in Figure S1).

### 3.2. DHA Induces RvD1 Production, a Metabolite Derived from DHA, in BEND Cells

**Figure 2:**
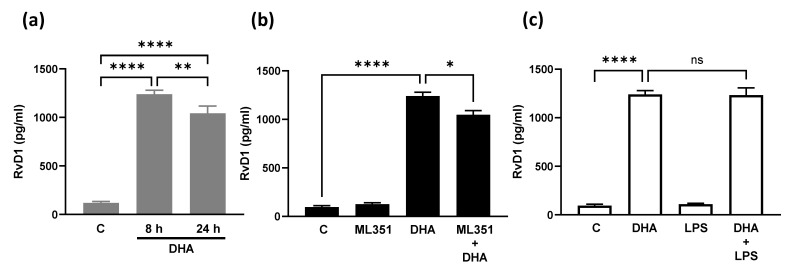
DHA induces resolvin D1 production, a metabolite derived from DHA, in BEND cells. (**a**) BEND cells were incubated with 50 μM DHA (or vehicle) for 8 or 24 h, and RvD1 levels in the culture medium were assessed using ELISA. (**b**) BEND cells were incubated with 5 μM ML351, and then 50 μM DHA (or vehicle) was added and incubated for 8 h. RvD1 in the culture medium was analyzed using ELISA. (**c**) BEND cells were incubated with 50 μM DHA (or vehicle), and then 500 ng/mL LPS (or vehicle) was added and incubated for 8 h. RvD1 in the culture medium was assessed using ELISA. C = vehicle. * *p* < 0.05, ** *p* < 0.01, **** *p* < 0.0001, ns = not significant.

We assess the production of RvD1 in BEND cells treated with DHA for 8 or 24 h. DHA significantly increased RvD1 production, a metabolite of DHA, at both 8 and 24 h, with slightly higher levels at 8 h (Figure 2a). BEND cells were incubated with the inhibitor of 15-Lipooxygenase (15-LO), ML351, and DHA for 8 h, and RvD1 production was assessed. ML351 significantly reduced DHA-induced RvD1 production (Figure 2b), suggesting the involvement of 15-LO in this effect, although this reduction did not reach the baseline level. To determine whether DHA-induced RvD1 production could be modified in the presence of LPS, BEND cells were incubated with DHA, and LPS was added and incubated for 8 h. The production of RvD1 was not modified in BEND cells treated with DHA and LPS compared to that with DHA (Figure 2c), highlighting the positive effect of DHA even in the presence of an inflammatory stimulus.

### 3.3. RvD1 Increases ERK1/2 and Akt Phosphorylation and Reduces LPS-Induced CXCL8 in BEND Cells

**Figure 3:**
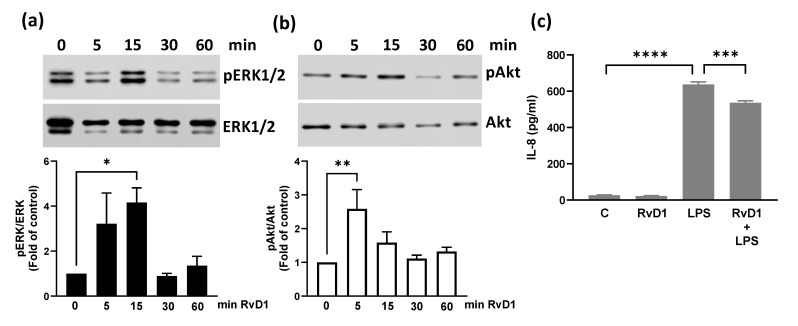
Resolvin D1 induced ERK1/2 and Akt phosphorylation and reduced CXCL8 production. BEND cells were incubated with 100 nM RvD1 (or vehicle) for different times. ERK1/2 (**a**) or Akt (**b**) phosphorylation was analyzed by immunoblotting with antibodies against phospho-ERK1/2 and total ERK1/2 and phospho-Akt and total Akt. C = vehicle. Bands’ intensities are shown as the ratio phospho-ERK1/2/ERK1/2 and phospho-Akt/Akt in the bar graph. Images are representative of three independent experiments. In (**c**), BEND cells were incubated with 100 nM DHA (or vehicle), and then 500 ng/mL LPS (or PBS) was added and incubated for 24 h. CXCL8 production was assessed in the culture medium by ELISA. * *p* < 0.05, ** *p* < 0.01, *** *p* < 0.001, **** *p* < 0.0001.

BEND cells were incubated with 100 nM RvD1 for different times, and immunoblotting for ERK1/2 and PI3K/Akt phosphorylation was performed. The results showed that RvD1 significantly increased the phosphorylation of ERK1/2 at 15 min (Figure 3a; whole blot images are shown in Figure S1) and Akt at 5 min (Figure 3b; whole blot images are shown in Figure S1). We also studied whether RvD1 affects LPS-induced CXCL8 production. For this, BEND cells were treated with RvD1 and LPS for 24 h, and CXCL8 production was determined. RvD1 significantly reduced LPS-induced CXCL8 production (Figure 3c).

### 3.4. Metabolomics Changes Induced by DHA in BEND Cells

**Figure 4:**
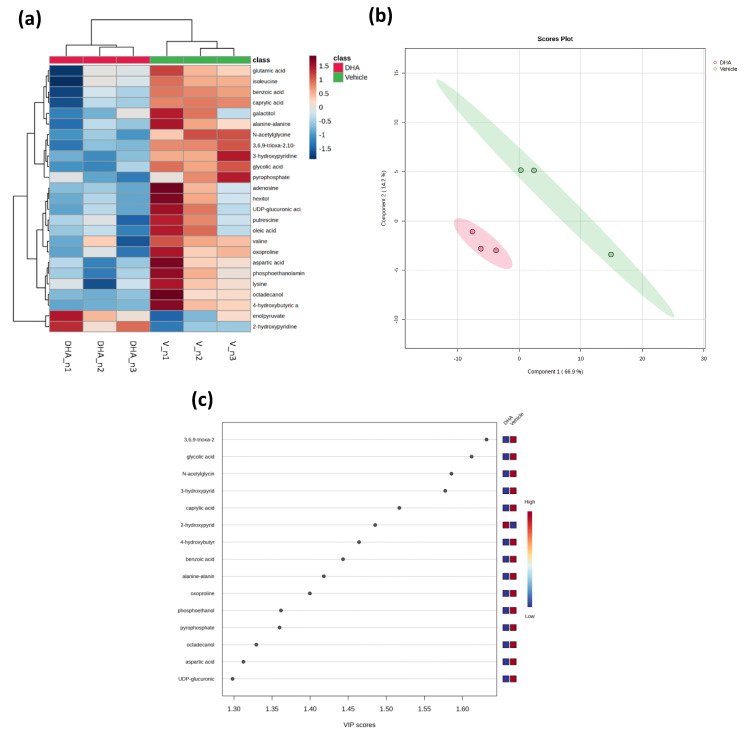
Metabolomic changes induced by DHA in BEND cells. (**a**) Heatmap of 25 metabolites with the lowest *p*-values from vehicle and DHA treatment. Metabolite levels that increased are shown in red and decreased in blue, compared to the mean metabolite relative abundance. (**b**) Partial Least Squares-Discriminant Analysis (PLS-DA) score plot based on the metabolomic analysis of the vehicle (green) and DHA treatment (red). (**c**) VIP score plot of the metabolites identified by PLS-DA in the vehicle and DHA treatments.

Metabolomics analysis of BEND cells treated with DHA compared to the vehicle identified 118 metabolites with different chemical entities. A heatmap plot of the 25 lowest *p*-values was generated to obtain a more intuitive visualization of the normalized metabolomic data (Figure 4a). The vehicle and DHA treatments showed a clear hierarchical separation, with metabolites belonging mainly to the amino acid and fatty acid groups exhibiting greater changes. It was observed that one metabolite increased after DHA treatment (2-hydroxypyridine). On the contrary, nine metabolites significantly decreased in the DHA treatment (3,6,9-trioxa-2,10-disilaundecane, 2,2,10,10-tetramethyl, glycolic acid, N-acetylglycine, 3-hydroxypyridine, caprylic acid, 4-hydroxybutyric acid, benzoic acid, alanine-alanine, and oxoproline). Partial least squares-discriminant analysis (PLS-DA) was used to discriminate between the vehicle and DHA treatment, with PC1 and PC2 values of 66.9% and 14.2% of the total variation, respectively (Figure 4b). The fold change (FC) and statistical significance (*p*-value) of the metabolites mentioned above between the vehicle and DHA treatments are visualized in Figure 1c (VIP score plot) and shown in Table 1. The metabolites with higher significance were 3,6,9-trioxa-2,10-disilaundecane, 2,2,10,10-tetramethyl, glycolic acid, N-acetylglycine, and 3-hydroxypyridine, which were reduced in the DHA treatment. By contrast, 2-hydroxypyridine significantly increased in the DHA treatment.

## 4. Discussion

This study showed that DHA reduced the production of pro-inflammatory mediators and promoted pro-resolution conditions in BEND cells. DHA reduced LPS-induced IL-6 and PTGS2 expression and increased RvD1 production. Interestingly, DHA increased LPS-induced CXCL8 production and did not modify RvD1 production in the presence of LPS, suggesting that DHA induces pro-resolution conditions without affecting the role of CXCL8 in innate immunity.

Regulation of endometrial immunity is a key process in post-calving cows because an adequate innate immune function and inflammatory response must be activated in the first days postpartum to induce defense mechanisms against pathogens and produce uterine involution [5]. However, the inflammatory response must be controlled to prevent chronic inflammation. We demonstrated in an in vitro model that DHA reduced LPS-induced IL-6 and PTGS2 expression stimulated by LPS in bovine endometrial cells. IL-6 is produced by several immune cell types, mainly macrophages and mast cells, but also by bovine endometrial cells [37]. In addition, IL-6 levels are increased in inflammatory diseases in ruminants, such as mastitis [46], ruminal acidosis [47], and metritis [48]. IL-6 is recognized as an inflammatory cytokine that contributes to the generation of a pro-inflammatory state. IL-6 induces the production of acute phase proteins (APP) from the liver, and APP has been identified in milk or serum in mastitis or metritis [49]. Therefore, the reduction of IL-6 production is important for decreasing the inflammatory response and diseases, such as metritis. Several studies have shown that DHA can reduce the production of pro-inflammatory mediators. In murine macrophages, DHA inhibits cytokine secretion, such as IL-6, through a mechanism involving the FFA4 receptor and the activation of β-arrestin2 [30]. In addition, DHA reduced LPS-induced IL-1 levels in THP-1 human monocytes [50]. PTGS2 is a key enzyme in inflammatory processes because it is involved in PGE2 synthesis. Previously, we demonstrated that DHA reduced LPS-induced PGE2 production in bovine endometrial cells [28]. DHA also inhibited PGE2 production and PTGS2 expression in murine macrophages [51] and cellular and animal models of endometrial cancer [52].

In contrast, our study showed that DHA increased LPS-induced CXCL8 expression in bovine endometrial cells. CXCL8 is a potent chemoattractant and activating factor for neutrophils, which are responsible for bacterial clearance [5,53]. Previous studies have shown that neutrophils function begins to decline before parturition and returns to prepartum levels around 4 weeks postpartum [54,55,56]. DHA could increase the bactericidal response because it stimulates ROS production and phagocytic activity in bovine, rat, and goat neutrophils [27,57,58], which is interesting because neutrophils can continue to destroy pathogens even in the presence of DHA, whereas a reduction in other pro-inflammatory cytokines occurs. The role of CXCL8 in the bovine reproductive tract has been evaluated through intrauterine or intravaginal administration of recombinant bovine CXCL8. The study of [59] showed that CXCL8 increased the proportion of neutrophils in the reproductive tract after 3 h of intravaginal administration in heifers or after 24 h of intrauterine administration in lactating cows, without adverse effects on health and welfare. When CXCL8 was administered by intrauterine infusion 24 h postpartum within multiparous dairy cows, it reduced the incidence of puerperal metritis and improved milk production and metabolic health [60]. Therefore, further studies on the effects of DHA-induced CXCL8 increases in bovine endometrial cells are necessary.

In addition to the effect of DHA on IL-6 and CXCL8 production, our study also showed that DHA reduced the phosphorylation of ERK1/2 and Akt induced by 15 and 60 min of LPS stimulation, respectively. ERK1/2 and Akt are two kinases that belong to the MAPK and PI3K/Akt pathways, respectively, and play crucial roles in various cellular processes. They are activated through signaling cascades by distinct inflammatory signals and are often involved in cell growth, differentiation, survival, and gene expression in immune cells and cancer [61,62]. In human endometrium, it has been shown that ERK1/2 is activated by different inflammatory stimuli [63,64]. The role of ERK1/2 in inflammation has been demonstrated in bovine endometrial cells using different compounds, such as melatonin or progesterone, which inhibited LPS-induced ERK1/2 phosphorylation and inflammatory cytokines, and melatonin reduced endometrial fibrosis, a condition associated with bovine endometritis [65,66]. Although DHA can reduce the phosphorylation of ERK1/2 or Akt induced by inflammatory stimuli, the underlying mechanism remains to be elucidated.

While DHA reduced LPS-induced ERK1/2 and Akt phosphorylation, RvD1 alone stimulated ERK1/2 and Akt phosphorylation. Our study showed that RvD1 induced rapid and transient ERK1/2 and Akt phosphorylation (15 and 5 min, respectively). This effect could be mediated through binding to the ALX/FPR2 or GPR32 receptors because RvD1 is recognized as a ligand for these receptors. Activation of ALX/FPR2 induces ERK1/2 phosphorylation and stimulates anti-inflammatory and pro-resolving signaling [67,68,69]. The activation of ERK1/2 and Akt by RvD1 has been linked to cell survival [70,71], and survival is critical in macrophage-mediated efferocytosis of apoptotic neutrophils, which represents another mechanism of resolution [72]. Resolvins play a role in the pro-resolution state, preventing chronic inflammation, and DHA is a precursor for RvD. In this study, we demonstrated that DHA induced RvD1 production, and the presence of LPS did not modify the RvD1 levels stimulated by DHA in bovine endometrial cells. The effects of resolvins on endometrial cells and inflammatory diseases have only been described in a rat endometriosis model, showing that the administration of RvD1 decreased vascular permeability in ectopic tissue, a sign of inflammation [73,74]. Furthermore, administration of omega-3 eicosapentaenoic acid (EPA), a precursor of E-series resolvins, in an endometriosis mouse model reduced the number of cystic lesions and expression of IL-6, and the 12/15-LOX pathway was key in the production of EPA metabolites for anti-inflammatory effects and suppression of endometriosis [75]. In addition, several studies have demonstrated the effects of RvD1 on macrophages and other cell types. RvD1 polarizes macrophages to an anti-inflammatory phenotype, modulating inflammation in adipocytes [76,77,78]. A growing body of evidence has led to resolution of inflammation to emerge as a promising therapeutic strategy, with a paradigm shift from conventional anti-inflammatory strategies to treatments focused on resolution to control chronic inflammatory disorders [78]. Diverse mechanisms by which RvD1 contributes to inflammation resolution have been proposed, such as inhibition of granulocyte recruitment to the inflammation site and pro-inflammatory signaling pathways, efferocytosis, and M1-to-M2 polarization [77,78,79,80]. In addition, therapeutic implications of SPMs have been proposed for disorders such as inflammatory bowel disease, osteoarthritis, and rheumatoid arthritis [78]. Therefore, all evidence supports the beneficial effects of SPM, such as RvD1, and compound precursors of RvD, such as DHA, in the prevention or treatment of inflammatory diseases.

Finally, our metabolomic assay showed a significant increase (2.7-fold) of a single metabolite, 2-hydroxypyridine, which can be found in equilibrium with 2-pyridone, and all other metabolites were slightly but significantly reduced after treatment of BEND cells with DHA. Studies suggest the effects that some of these metabolites could have; it has been shown that natural or synthetic derivatives of the 2-pyridone nucleus have anti-inflammatory activity in vivo in a mouse inflammation model [81] and in vitro in RAW264.7 macrophages [82]. 3-hydroxypyridine derivatives have also shown anti-inflammatory activity in a rat inflammation model and in human uterine chronic inflammation [83,84]. Caprylic acid (or octanoic acid) and benzoic acid could be beneficial for embryo development in mice [85,86]. However, studies have suggested that caprylic acid must be monitored and reduced because high concentrations can be embryotoxic and induce oxidative damage and disruption of mitochondrial bioenergetics [87,88]. N-acetylglycine has been suggested to modulate adipose tissue gene expression in obesity-associated pathways, such as immune response, lysosome function, and tissue remodeling, and may have a protective role against body fat accumulation [89,90]. Oxoproline is an intermediate of glutathione metabolism that induces protein oxidation and reactive species production [91]. Although our results from the metabolomics approach in bovine endometrial cells showed, for the first time, DHA-induced metabolomic changes in these cells, more detailed studies on pathway analysis and the effects of metabolites on the immune response in endometrial cells are needed.

## 5. Conclusions

Our study showed that DHA reduced LPS-induced PTGS2 and IL-6 expression and ERK1/2 and Akt phosphorylation induced by LPS. In addition, DHA stimulated RvD1 production, a metabolite derived from DHA, and RvD1 induced rapid ERK1/2 and Akt phosphorylation and reduced CXCL8 production. By contrast, DHA increased LPS-induced CXCL8, which needs to be studied; however, it could play a role in the innate defense mechanisms of phagocytes. Finally, DHA induced metabolomic changes, with one metabolite increasing and nine decreasing; however, its effects on endometrial cells need to be studied.

## Figures and Tables

**Table 1:** Metabolite changes in BEND cells treated with DHA. Fold changes (FC) and *p*-values (raw.pval) are shown for each metabolite.

Metabolite	FC	log2(FC)	raw.pval	“=−LOG10(p)”
3,6,9-trioxa-2,10-disilaundecane, 2,2,10,10-tetramethyl-	0.02884	−5.1158	0.0014157	2.849
glycolic acid	0.080281	−3.6388	0.0026738	2.5729
N-acetylglycine	0.0054968	−7.5072	0.0049903	2.3019
3-hydroxypyridine	0.015087	−6.0505	0.0058485	2.233
caprylic acid	0.021782	−5.5207	0.014175	1.8485
2-hydroxypyridine	2.7659	1.4677	0.019969	1.6996
4-hydroxybutyric acid	0.088814	−3.4931	0.024361	1.6133
benzoic acid	0.030144	−5.052	0.029112	1.5359
alanine-alanine	0.032934	−4.9243	0.035359	1.4515
oxoproline	0.016739	−5.9007	0.040257	1.3952

## Data Availability

The original contributions presented in this study are included in the article/Supplementary Material. Further inquiries can be directed to the corresponding author.

## Supplementary Materials

The following supporting information can be downloaded at: https://www.mdpi.com/article/10.3390/ani15172545/s1, Figure S1: whole blot images.

## Abbreviations

The following abbreviations are used in this manuscript:

DHA	Docosahexaenoic acid
BEND	Bovine endometrial
PTGS2	Prostaglandin-endoperoxide synthase 2
IL	Interleukin
RvD1	Resolvin D1
LPS	Lipopolysaccharide
ELISA	Enzyme-linked immunosorbent assay
RT-qPCR	Reverse transcription-quantitative polymerase chain reaction
ERK1/2	Extracellular signal-regulated kinase 1/2
NEFA	Non-esterified fatty acids
NEB	Negative energy balance
PUFA	Polyunsaturated fatty acid
TNF-α	Tumor necrosis factor-alpha
MMP-9	Mmatrix metalloproteinase-9
ROS	Reactive oxygen species
PGE2	ProstaglandinE2
SPM	Specialized pro-resolving mediators
FFA4	Free Fatty Acid-4
15-LO	15-lipoxygenase
GC-MS	Gas chromatography–mass spectrometry
HPLC	High performance liquid chromatography
