# Supplementary figures and images for: Docosahexaenoic Acid (DHA) Decreases IL-6 and Prostaglandin-Endoperoxide Synthase 2 mRNA Expression and IL-6 Protein Release, While Increasing Resolvin D1 and CXCL8 mRNA Expression and Protein Release in BovineEndometrial Cells
Source: Animals (Basel). 2025 Aug 29;15(17):2545. doi: 10.3390/ani15172545 (PMC12427235; doi:10.3390/ani15172545)

Figure 1

- pERK1/2, ERK1/2

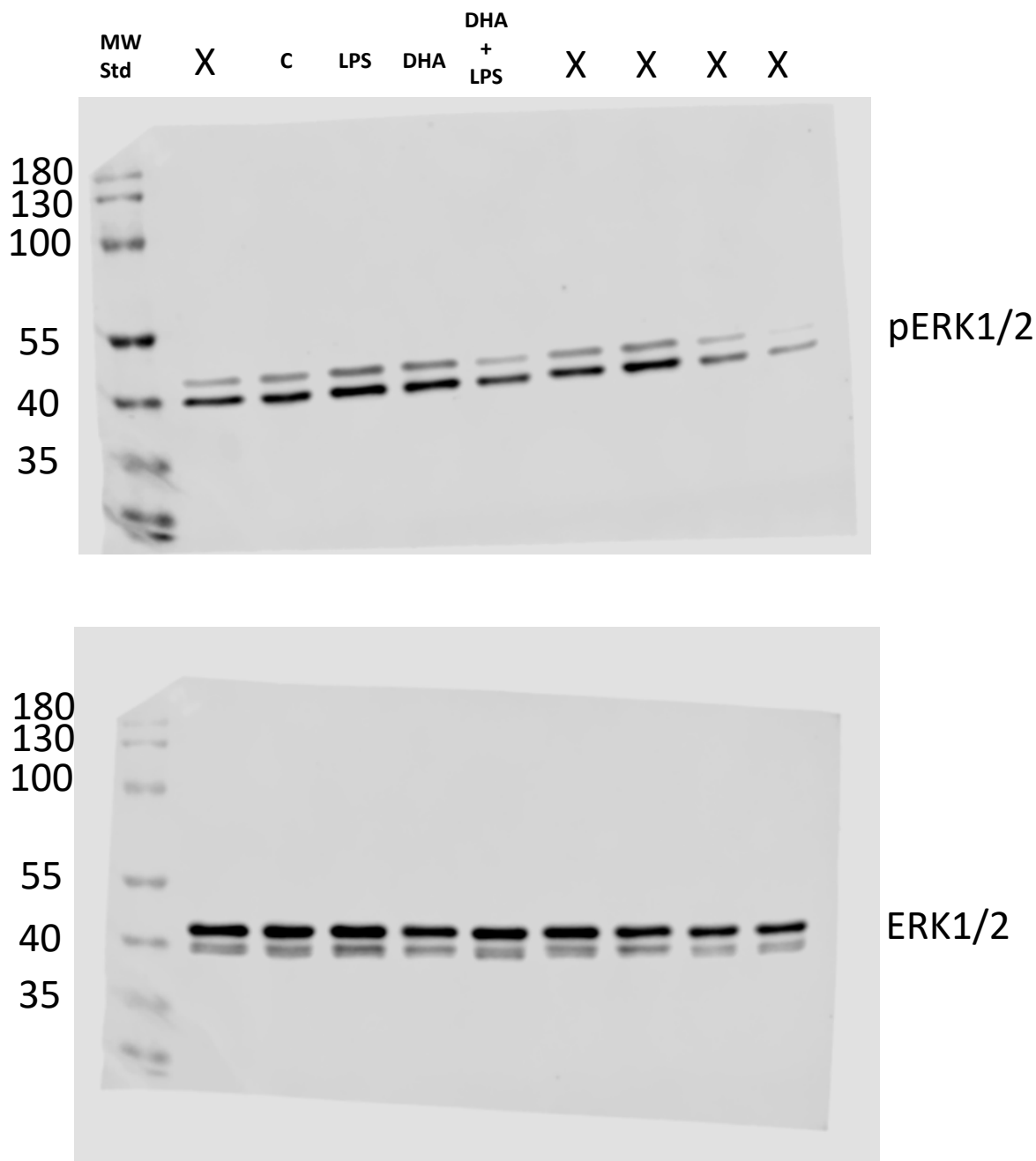

Figure 1

- pAkt, Akt

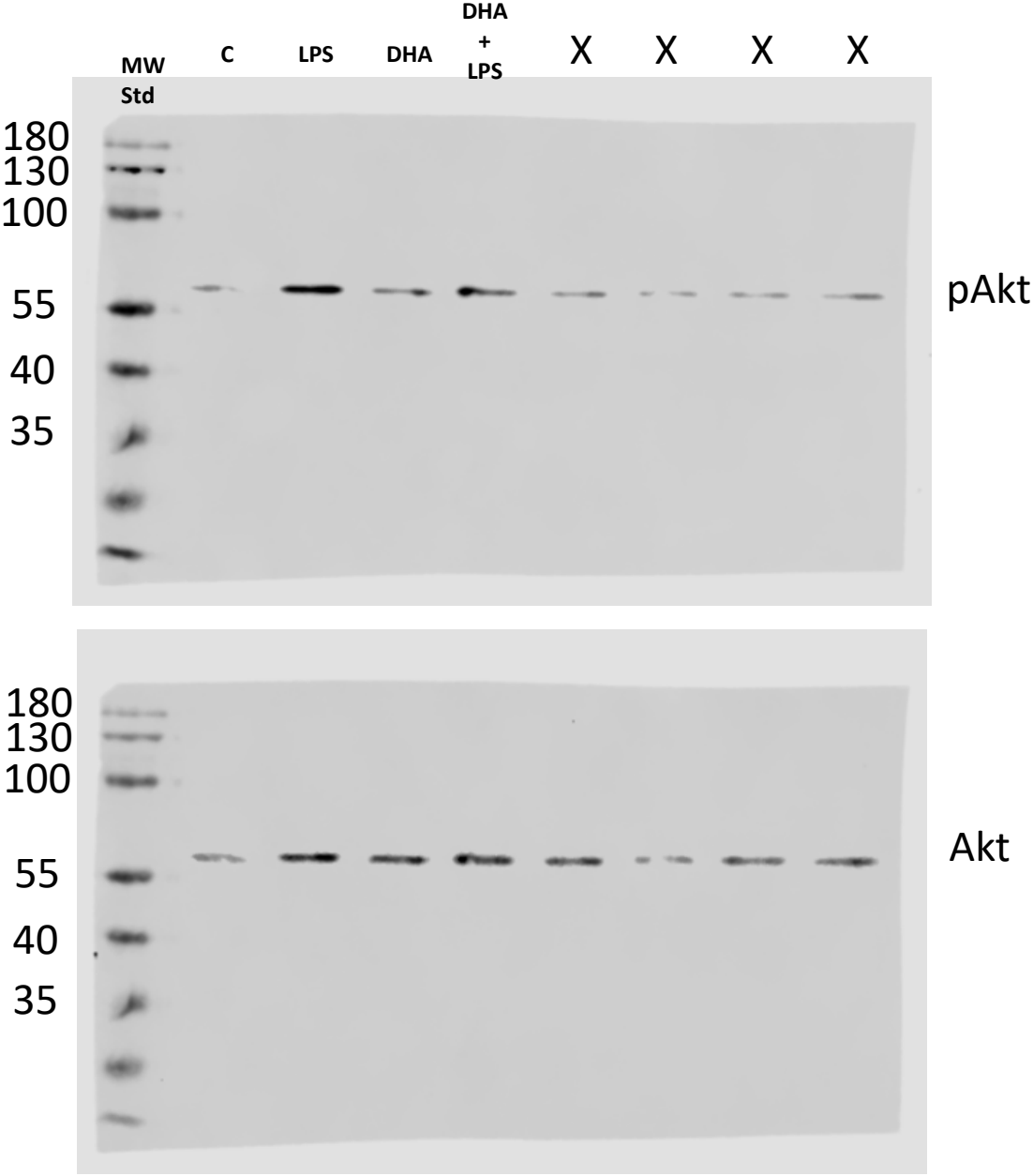

Figure 3

- pERK1/2, ERK1/2

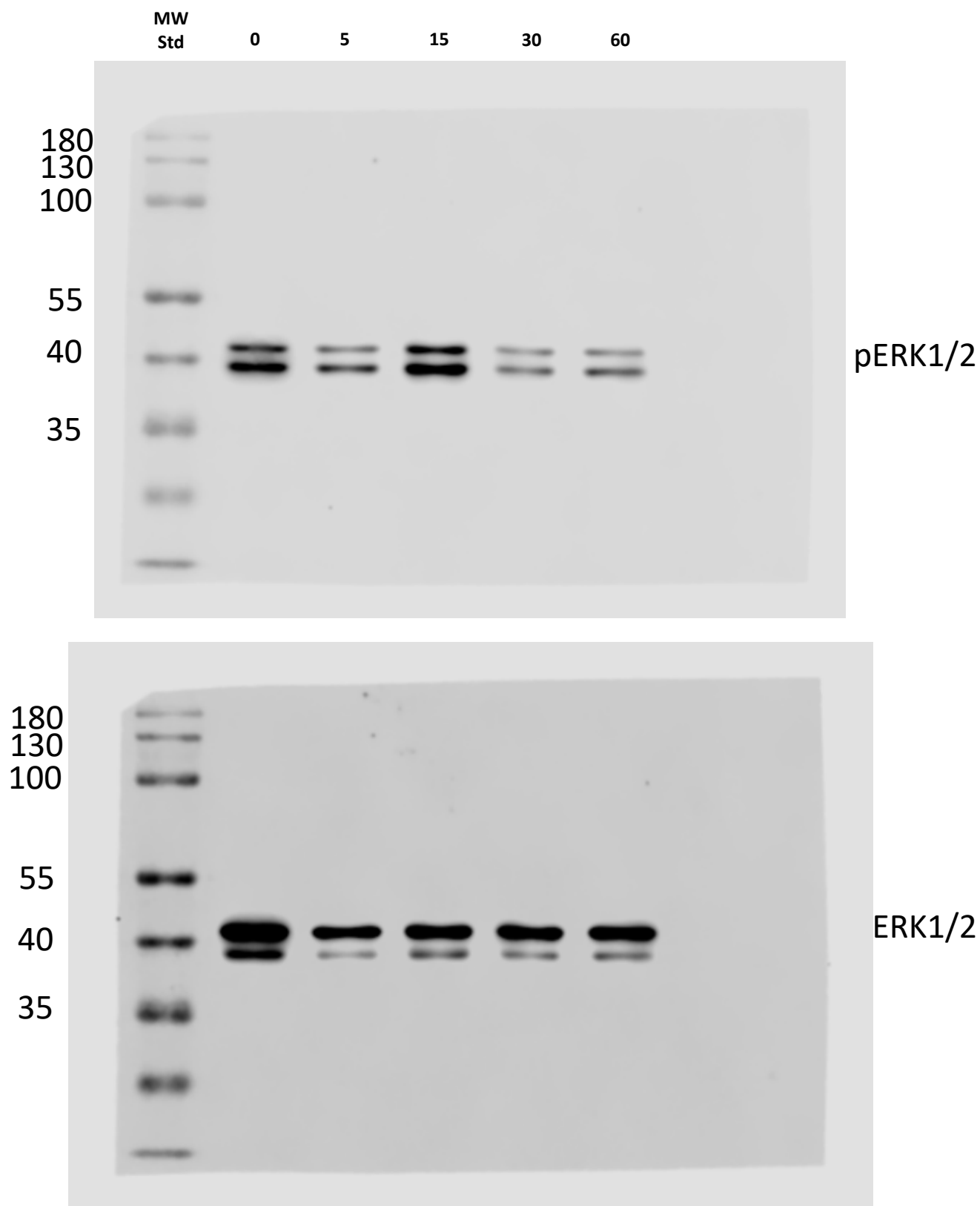

Figure 3

- pAkt, Akt

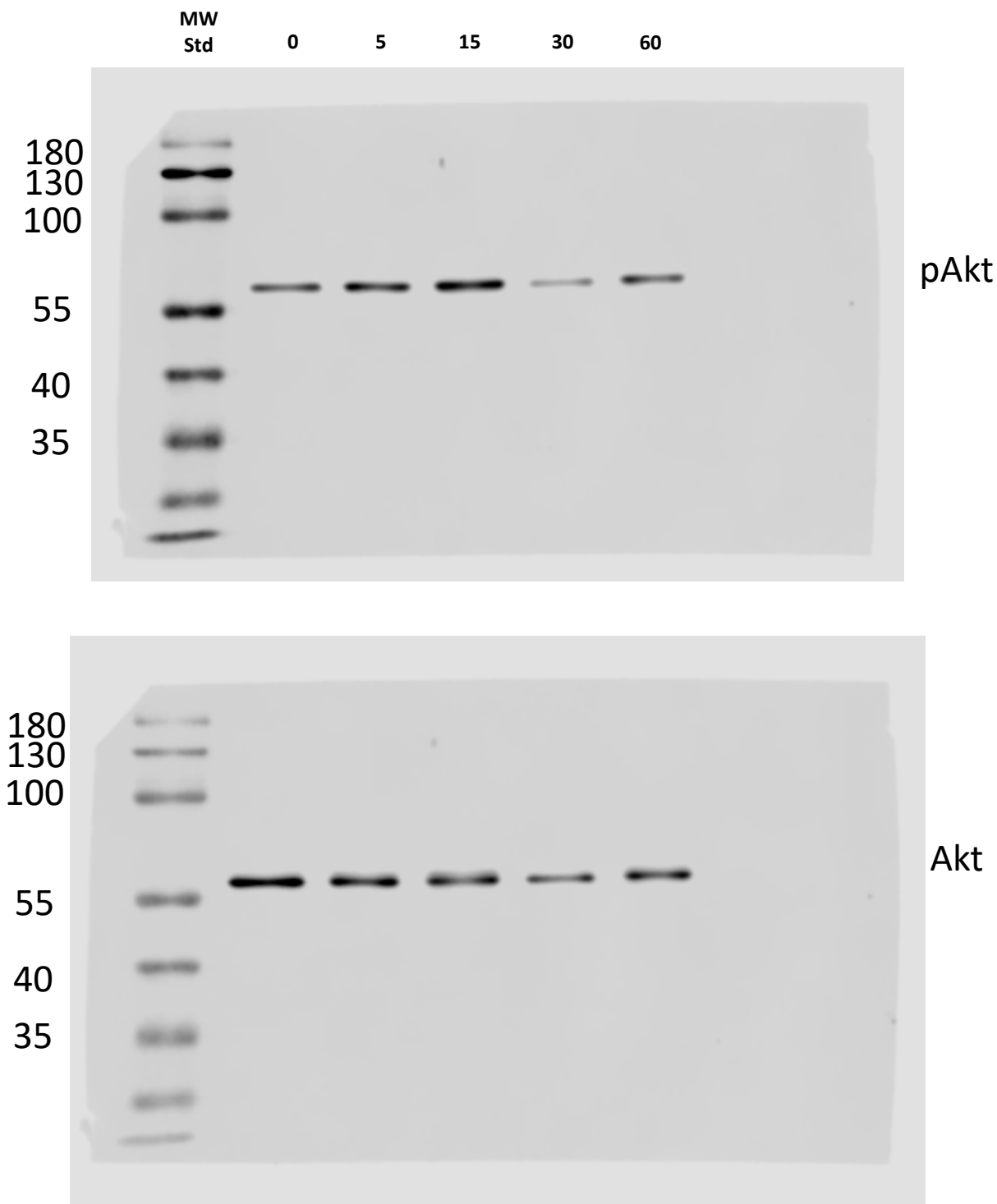

Supplement: Supplementary file 1 [file animals-15-02545-s001.zip › animals-3796875-supplementary.pdf]
